# The role of only-child status in the effect of childhood trauma and parental rearing style on depressive symptoms in Shanghai adolescents

**DOI:** 10.3389/fpsyt.2023.1196569

**Published:** 2023-06-21

**Authors:** Yingyan Zhong, Xinxin Huang, Jianhua Chen, Yuting Li, Yan Li, Rumeng Chen, Enzhao Cong, Yifeng Xu

**Affiliations:** ^1^Shanghai Mental Health Center, Shanghai Jiao Tong University School of Medicine, Shanghai, China; ^2^Shanghai Tenth People's Hospital, School of Medicine, Tongji University, Shanghai, China

**Keywords:** only-child status, depressive symptoms, childhood trauma, parental rearing style, emotional abuse, rejection

## Abstract

**Introduction:**

After decades of the one-child policy, China changed its rules to allow two children in 2016, which altered family dynamics. Few studies have examined the emotional problems and the family environment of multi-child adolescents. This study aims to explore the role of only-child status in the impact of childhood trauma and parental rearing style on depressive symptoms of adolescents in Shanghai, China.

**Methods:**

A cross-sectional study was conducted on 4,576 adolescents (*M* = 13.42 years, SD = 1.21) from seven middle schools in Shanghai, China. Childhood Trauma Questionnaire-Short Form, the Short Egna Minnen Beträffande Uppfostran, and Children’s Depression Inventory were used to evaluate childhood trauma, perceived parental rearing style, and depressive symptoms of adolescents, respectively.

**Results:**

Results showed that girls and non-only children reported more depressive symptoms, while boys and non-only children perceived more childhood trauma and negative rearing styles. Emotional abuse, emotional neglect, and father’s emotional warmth significantly predicted depressive symptoms in both only children and non-only children. Father’s rejection and mother’s overprotection were related to adolescents’ depressive symptoms in only-child families, but not non-only child families.

**Discussion:**

Therefore, depressive symptoms, childhood trauma, and perceived negative rearing styles were more prevalent among adolescents in non-only child families, while negative rearing styles were especially associated with depressive symptoms in only children. These findings suggest that parents pay attention to their impacts on only children and give more emotional care to non-only children.

## 1. Introduction

Researches in China and other countries have shown that depressive symptoms were increasing in adolescents (1–3). A recent survey of secondary school students in China showed that the pooled prevalence of depressive symptoms in mainland China was 24.3%, which was higher than the prevalence in the general population (4). Adolescent depression increases the risk for depression and other psychosocial dysfunctions in adulthood (5), which deserves public attention.

The reasons for adolescents’ depressive symptoms are multifaceted, including personality factors, social support, coping strategies, and negative life events (6), among which family factors might be the most essential. Previous studies showed that low family support (7, 8), negative parenting styles (9), and distant parental-child relationships (10) were associated with high levels of adolescent depressive symptoms. Parental rearing style is a constellation of stable attitudes expressed by parents through specific, goal-directed behaviors (such as parenting practices) and non-goal-directed behaviors (such as emotional expression) to communicate and interact with their children (11), which is a significant predictor of adolescent mental health problems (12). It includes three dimensions (rejection, emotional warmth, and overprotection) according to the Short Egna Minnen Beträffande Uppfostran which was widely used in research (13). Negative parental rearing styles like parental rejection positively predicted depressive symptoms in adolescents (14–16). However, parental warmth was a protective factor for adolescent depression, which predicted a lower degree of depression (17).

Moreover, negative parental rearing styles were related to cause childhood trauma in adolescents (14). According to the literature on clinical psychology (18, 19), childhood trauma refers to a scary, dangerous, violent, or life-threatening event that happens to a child, which leads to lots of mental health problems in adolescents such as depression and anxiety. Bernstein and colleagues divided childhood trauma into five categories: emotional abuse (EA), physical abuse (PA), sexual abuse (SA), emotional neglect (EN), and physical neglect (PN) (20). All these types of childhood trauma were related to adolescents’ depressive symptoms (21). Notably, as emotional trauma was more subtle, imperceptible, and lasting, emotional types of traumas had the strongest associations with depressive symptoms (22, 23). Parental emotional neglect and abuse in childhood were associated with the formation of insecure attachments and emotional regulation strategies, which develop more depressive symptoms (24).

Following China’s relaxation of its over three-decade-long family planning policy and adoption of the universal two-child policy in 2016, the family structure has transformed from one-child families into two-child or multi-child families. The differences in family atmosphere and parenting styles have caught the attention of researchers in both types of families (25, 26). Only-child families had a more positive family atmosphere and the members expressed their emotions more directly to each other which was reflected in the fact that the intimacy between only children and their parents was higher than that of non-only children (25). In only-child families, parents tended to adopt positive parenting styles and provide more emotional care and love to their children (26). The mental health of only children was more susceptible to negative parenting styles (27, 28). In contrast, non-only children had more traumatic childhood experiences and exhibited more depressive symptoms (29). However, previous studies mostly assessed parental rearing styles in general (9, 14), but it was proved that fathers and mothers had different emotional effects on their children (12). Moreover, most studies on adolescents’ depressive symptoms did not take the only-child status into consideration. More researches are needed to explore the emotional problems and family factors of adolescents in multi-child families.

To further explore parental influences on adolescents’ depressive symptoms, this study investigated all five forms of childhood trauma and three dimensions of parental rearing styles in the father and mother separately. We hypothesize that (1) non-only children exhibit more depressive symptoms than only children. (2) Emotional types of childhood trauma (emotional abuse and neglect) are greater predictors of depressive symptoms in adolescents. (3) Negative parental rearing styles have greater impacts on depressive symptoms of only children than non-only children.

## 2. Methods

### 2.1. Participants and procedure

This cross-sectional online survey was conducted from September 23rd, 2021 to October 16th, 2021 in Shanghai, China. Participants from seven different junior high schools (grades 6–9) participated in the survey. The questionnaires were uniformly sent by “Questionnaire Star” online (Questionnaire Star: an online questionnaire platform). School psychologists and head teachers guided adolescents to complete the questionnaires. Before the survey, adolescents were informed that all the content filled in was confidential. Parents were also informed about the nature of the survey and their consent was obtained. After completing the questionnaire, feedback on research findings was provided for the school psychologists. Additionally, researchers are committed to assisting them in implementing necessary interventions for adolescents within relevant psychology courses. The research was officially approved by the IRB of Shanghai Mental Health Center (Ethics Approval Number: 2021–11).

In total, 4,888 questionnaires were collected. After excluding the invalid questionnaires (repeatedly filled questionnaires, filling times were beyond three standard deviations of the average, and those ages exceeded the required standard of the survey), the number of remaining valid questionnaires was 4,576. After obtaining valid questionnaires, personal information including name and school was anonymized. A unique sequence number was assigned to each student, and a numerical code was generated for each school to ensure confidentiality and protect the participants’ privacy. The sample was aged from 9 to 17 years (*M* = 13.42 years, *SD* = 1.21). Approximately half of them were boys (2,325, 50.81%) and half were girls (2,251, 49.19%). A total of 3,236 (70.72%) adolescents were only-child. Among all adolescents, 1,378 (30.11%) were sixth-grade students, 1,453 (31.75%) were seventh-grade students, 1,107 (24.19%) were eighth-grade students and 638 (13.94%) were ninth-grade students.

### 2.2. Measures

#### 2.2.1. Childhood trauma

Childhood Trauma Questionnaire-Short Form (CTQ-SF) is a 28-item self-report questionnaire to measure adolescents’ trauma experiences in childhood (20, 30). The questionnaire contains five forms of abuse or neglect: emotional abuse (EA), physical abuse (PA), sexual abuse (SA), emotional neglect (EN), and physical neglect (PN). Items are rated on a 5-point Likert scale, ranging from 1 (*never true*) to 5 (*very often*). Higher scores represent more perceived traumatic events in childhood. The Cronbach’s α was 0.65 in the current study.

#### 2.2.2. Parental rearing style

The adolescent version of the Short Egna Minnen Beträffande Uppfostran (s-EMBU) was a self-report questionnaire to assess perceived parental rearing style which directly affected adolescents (31, 32). The 21-item scale has three subscales: rejection, emotional warmth, and overprotection. Items are rated on a 4-point Likert scale ranging from 1 (*never*) to 4 (*always*). Adolescents marked each item for both their mother’s and father’s rearing styles. The higher the score on the scale, the more adolescents perceive this type of parenting style. The scores of the mother and father scales were separately calculated. The Cronbach’s α was 0.71 (father) and 0.73 (mother) in the current study.

#### 2.2.3. Depressive symptoms

Children’s Depression Inventory (CDI) was administered to assess adolescents’ depressive symptoms (33). The scale is applicable to measure depressive symptoms of minors aged 7–17, with a total of 27 items. Each item has three different levels of options to describe the relevant symptoms. Adolescents were asked to rate each item based on a 3-point scale (0–2). An example item is “I am sad once in a while” (0), “I am sad many times” (1), and “I am sad all the time” (2). A higher score indicates more severe depressive symptoms and a score of 19 is classified as clinical depression. The Cronbach’s α was 0.90 in the current study.

#### 2.2.4. Statistical analysis

Statistical analysis was performed in SPSS version 22.0. Descriptive statistics were reported as mean ± standard (*SD*) or frequency (percentage). One-way analysis of variance (ANOVA), *post hoc* multiple comparisons and independent *t*-tests were used to compare the scores of depressive symptoms with different characteristics. The analysis of variance was used to compare the difference between boys/girls and only/non-only children in different measures. Hierarchical linear regression using the “enter” method was conducted with significant variables of correlation analysis as independent variables and depressive symptoms as the dependent variables. In step 1, demographic controls (gender, grade and parent’s education levels) were entered. In step 2, childhood trauma and parental rearing style were entered as predictors. Furthermore, to explore whether there were only-child/non-only-child differences in the effects of childhood trauma and parental rearing style on adolescent depressive symptoms, only-child and non-only-child families were analyzed separately. In all regression, the values of the variance inflation factor (VIF) were less than 10, ranging from 1.00 to 5.29. The significance value was set at *p* < 0.05 (two-tailed) in this study.

## 3. Results

### 3.1. Descriptive analysis

Out of the total number of 4,576 valid participants, the prevalence of clinical depression (CDI ≥ 19) is 13.77%. Table 1 showed the descriptive statistics and scores of depressive symptoms with different characteristics within the sample. Girls (*t* = −4.48, *p* < 0.01) and non-only children (*t* = 5.07, *p* < 0.01) reported significantly more depressive symptoms compared to boys and only children. One-way ANOVAs and *post hoc* multiple comparisons showed that those of high grades [*F*(3, 4,572) = 2.82, *p* < 0.05] and low parents’ education level [father: *F*(3, 4,572) = 41.39, *p* < 0.01; mother: *F*(3, 4,572) = 32.36, *p* < 0.01] were more likely to feel depressed.

**Table 1:** Descriptive statistics and scores of depressive symptoms in different characteristics of adolescents.

Variables	M ± SD/*n* (%)	Scores of depressive symptoms	*F*/*t*	*p*
**Age (years)**	13.42 ± 1.21	10.41 ± 7.44		
**Gender**			−4.48	0.00**
Boys	2,325 (50.81)	9.93 ± 6.85		
Girls	2,251 (49.19)	10.92 ± 7.98		
**Grade**			2.82	0.04*
Grade 6	1,378 (30.11)	9.95 ± 6.99		
Grade 7	1,453 (31.75)	10.51 ± 7.68		
Grade 8	1,107 (24.19)	10.74 ± 7.81		
Grade 9	638 (13.94)	10.64 ± 7.16		
**Whether the only-child**			5.07	0.00**
No	1,340 (29.28)	11.30 ± 7.73		
Yes	3,236 (70.72)	10.05 ± 7.29		
**Father’s education level**			41.39	0.00**
Graduate degree or above	464 (10.14)	9.50 ± 7.16		
Undergraduate	2,595 (56.71)	9.60 ± 6.93		
Senior middle school or Technical secondary school	1,094 (23.91)	11.75 ± 8.13		
Junior high school and below	423 (9.24)	12.92 ± 7.85		
**Mother’s education level**			32.36	0.00**
Graduate degree or above	324 (7.08)	9.85 ± 7.37		
Undergraduate	2,876 (62.85)	9.74 ± 7.13		
Senior middle school or Technical secondary school	866 (18.92)	11.47 ± 7.71		
Junior high school and below	510 (11.15)	12.79 ± 8.02		

^*^p < 0.05. ^**^p < 0.01.

### 3.2. Gender differences and only-child status differences

Table 2 presented the differences in childhood trauma, parental rearing style, and depressive symptoms between boys/girls as well as only/non-only children. The results showed that gender and only-child status play an important role in emotional abuse [*F*(3, 4,572) = 4.38, *p* < 0.01], physical abuse [*F*(3, 4,572) = 11.80, *p* < 0.01], sexual abuse [*F*(3, 4,572) = 2.86, *p* < 0.05], emotional neglect [*F*(3, 4,572) = 13.27,*p* < 0.01], physical neglect [*F*(3, 4,572) = 6.98, *p* < 0.01], father’s rejection [*F*(3, 4,572) = 9.31, *p*< 0.01], mother’s rejection [*F*(3, 4,572) = 3.72, *p* < 0.05], emotional warmth from father (*F* = 13.95, *p* < 0.01), emotional warmth from mother [*F*(3, 4,572) = 16.67, *p* < 0.01], father’s overprotection [*F*(3, 4,572) = 3.03, *p* < 0.05] and depressive symptoms [*F*(3, 4,572) = 15.22, *p* < 0.01]. The results of independent *t*-tests for gender and only child status are listed in Supplementary Table S1.

**Table 2:** Gender and only-child/non-only-child differences in childhood trauma, parental rearing style, and depressive symptoms.

	Total (*n* = 4,576)	Only-child boys (*n* = 1,696)	Only-child girls (*n* = 1,540)	Non-only-child boys (*n* = 629)	Non-only-child girls (*n* = 711)	*F*	*p*
EA	6.70 ± 2.48	6.58 ± 2.35	6.73 ± 2.47	6.66 ± 2.49	6.98 ± 2.77	4.38	0.00**
PA	5.54 ± 1.56	5.64 ± 1.70	5.36 ± 1.21	5.71 ± 1.80	5.56 ± 1.65	11.80	0.00**
SA	5.11 ± 0.87	5.14 ± 1.00	5.07 ± 0.55	5.17 ± 1.18	5.11 ± 0.81	2.86	0.04*
EN	8.93 ± 3.66	8.78 ± 3.59	8.65 ± 3.58	9.48 ± 3.82	9.42 ± 3.74	13.27	0.00**
PN	6.86 ± 2.37	6.82 ± 2.34	6.69 ± 2.27	7.09 ± 2.45	7.09 ± 2.56	6.98	0.00**
Rejection (*F*)	7.99 ± 2.68	8.11 ± 2.71	7.75 ± 2.51	8.35 ± 2.92	7.94 ± 2.68	9.31	0.00**
Rejection (*M*)	8.03 ± 2.65	8.03 ± 2.62	7.88 ± 2.57	8.15 ± 2.66	8.24 ± 2.90	3.72	0.01*
Emotional warmth (*F*)	22.15 ± 4.90	22.50 ± 4.70	22.40 ± 4.82	21.48 ± 5.18	21.39 ± 5.14	13.95	0.00**
Emotional warmth (*M*)	22.74 ± 4.54	23.09 ± 4.40	22.98 ± 4.32	22.21 ± 4.82	21.87 ± 4.92	16.67	0.00**
Overprotection (*F*)	15.72 ± 4.05	15.91 ± 3.90	15.50 ± 4.17	15.82 ± 4.09	15.64 ± 4.08	3.03	0.03*
Overprotection (*M*)	16.37 ± 4.22	16.52 ± 3.94	16.21 ± 4.45	16.25 ± 4.18	16.42 ± 4.39	1.65	0.18
Depressive symptoms	10.41 ± 7.44	9.66 ± 6.78	10.48 ± 7.79	10.67 ± 6.99	11.86 ± 8.30	15.22	0.00**

EA, emotional abuse; PA, physical abuse; SA, sexual abuse; EN, emotional neglect; PN, physical neglect; F, father; M, mother. ^*^p < 0.05. ^**^p < 0.01.

### 3.3. Hierarchical linear regression analysis

Based on the correlation among childhood trauma, parental rearing style, and depressive symptoms (see Supplementary Table S2), hierarchical linear regression analysis was conducted. As depicted in Tables 3, 4, we examined the effects of childhood trauma and parental rearing style on depressive symptoms in only children and non-only children respectively, controlling for gender, grade and parents’ education levels. For only children (*n* = 3,236, Δ*R^2^* = 0.447, *p* < 0.01), results indicated that emotional abuse (*β* = 0.28, *p* < 0.01), emotional neglect (*β* = 0.07, *p* < 0.01), father’s rejection (*β* = 0.08, *p* < 0.01) and mother’s overprotection (*β* = 0.07, *p* < 0.05) were significant positive predictors of adolescents’ depressive symptoms, while emotional warmth from father (*β* = −0.12, *p* < 0.01) and mother (*β* = −0.10, *p* < 0.01) were significantly negative predictors. For non-only children (*n* = 1,340, Δ*R*^2^ = 0.426, *p* < 0.01), only the emotional dimension of childhood trauma and parental rearing style showed effects on their depressive symptoms. Higher emotional abuse (*β* = 0.24, *p* < 0.01) and emotional neglect (*β* = 0.14, *p* < 0.01) predicted more depressive symptoms. The more emotional warmth from their father that adolescents perceived, the fewer depressive symptoms they reported (*β* = −0.23, *p* < 0.01).

**Table 3:** Hierarchical regression analysis of childhood trauma, parental rearing style on depressive symptoms (only children, *n* = 3,236).

	Depressive symptoms					
	*b*	SE	*β*	*p*	Δ*R*^2^	Δ*F*
**Step 1**					0.023	19.258
Gender	0.77	0.25	0.05	0.00**		
Grade	0.13	0.13	0.02	0.32		
Father’s educational level	1.19	0.23	0.12	0.00**		
Mother’s educational level	0.34	0.23	1.49	0.14		
**Step 2**					0.447	246.871
Gender	0.87	0.19	0.06	0.00**		
Grade	0.07	0.09	0.01	0.44		
Father’s educational level	0.48	0.17	0.05	0.00**		
Mother’s educational level	0.01	0.17	0.00	0.96		
EA	0.85	0.05	0.28	0.00**		
PA	− 0.02	0.08	− 0.00	0.83		
SA	0.25	0.13	0.03	0.06		
EN	0.29	0.04	0.14	0.00**		
PN	0.12	0.05	0.04	0.01**		
Rejection (*F*)	0.21	0.07	0.07	0.00**		
Rejection (*M*)	0.14	0.08	0.05	0.07		
Emotional warmth (*F*)	− 0.18	0.05	− 0.12	0.00**		
Emotional warmth (*M*)	− 0.17	0.05	− 0.10	0.00**		
Overprotection (*F*)	0.06	0.05	0.03	0.23		
Overprotection (*M*)	0.12	0.05	0.07	0.02*		

EA, emotional abuse; PA, physical abuse; SA, sexual abuse; EN, emotional neglect; PN, physical neglect; F, father; M, mother. ^*^p < 0.05. ^**^p < 0.01.

**Table 4:** Hierarchical regression analysis of childhood trauma, parental rearing style on depressive symptoms (non-only children, *n* = 1,340).

	Depressive symptoms					
	*b*	SE	*β*	*p*	Δ*R*^2^	Δ*F*
**Step 1**					0.035	12.025
Gender	1.20	0.42	0.08	0.00**		
Grade	0.08	0.21	0.01	0.72		
Father’s educational level	0.62	0.34	0.07	0.07		
Mother’s educational level	0.92	0.34	0.11	0.01**		
**Step 2**					0.426	95.120
Gender	0.96	0.32	0.06	0.00**		
Grade	− 0.27	0.16	− 0.04	0.08		
Father’s educational level	0.14	0.26	0.02	0.59		
Mother’s educational level	0.18	0.26	0.02	0.48		
EA	0.69	0.09	0.24	0.00**		
PA	− 0.06	0.13	− 0.01	0.60		
SA	0.22	0.18	0.03	0.23		
EN	0.29	0.06	0.14	0.00**		
PN	0.10	0.07	0.03	0.22		
Rejection (*F*)	0.13	0.11	0.05	0.24		
Rejection (*M*)	0.11	0.11	0.04	0.33		
Emotional warmth (*F*)	− 0.35	0.07	− 0.23	0.00**		
Emotional warmth (*M*)	− 0.10	0.07	− 0.06	0.17		
Overprotection (*F*)	0.11	0.09	0.06	0.21		
Overprotection (*M*)	0.13	0.08	0.07	0.11		

EA, emotional abuse; PA, physical abuse; SA, sexual abuse; EN, emotional neglect; PN, physical neglect; F, father; M, mother. ^*^p < 0.05. ^**^p < 0.01.

## 4. Discussion

The main finding of this research was that in Chinese adolescents, girls and non-only children reported more depressive symptoms, while boys and non-only children perceived more childhood trauma and negative rearing styles. All types of childhood trauma and parental rearing styles were related to adolescents’ depressive symptoms. In particular, emotional abuse and neglect significantly predicted depressive symptoms in both only-child and non-only-child families. Father’s rejection and mother’s overprotection were positive predictors of depressive symptoms in only children. Father’s emotional warmth was the protective factor against depressive symptoms in both only children and non-only children.

The survey revealed that girls reported more depressive symptoms than boys, which was in line with a prior study (2). Results showed that girls reported more emotional abuse, while boys experienced more physical abuse and sexual abuse. Moreover, boys reported more fathers’ rejection and overprotection. As a previous study showed, the gender of parents and children both had an impact on parental rearing styles (34). Fathers pay more attention to the cultivation of children’s autonomy, while mothers provide more emotional care and warmth. For boys, parents may be sterner, more refuse and ignore, and fathers are more willing to participate in the upbringing of boys (35). However, parents prefer to be tolerant of girls. From the perspective of adolescents, girls are more sensitive to emotional change than boys, which may lead to emotional overreaction and increase the risk of depressive symptoms (36).

Similar to prior researches (37, 38), this study found that only children reported fewer depressive symptoms than non-only children. Moreover, the results showed that only children perceived more emotional warmth from parents, while non-only children perceived more childhood trauma and parents’ rejection. Being the only children in the family allows he/she to receive more emotional support and attention from his/her parents, which helps he/she develop a stronger sense of confidence and consequently develop less depressive symptoms (37, 39). On the contrary, in multi-child families, parents face the challenge of allocating their limited resources such as money, time, and energy among their children, which makes it difficult to meet the different physical and emotional needs of each child. Thus, non-only children may experience more childhood trauma and negative rearing styles, especially emotional abuse (40). Parents’ negative rearing styles were related to adolescents’ early maladaptive schemas. Adolescents are more likely to form unsafe attachment styles and are easy to have a sense of self worthless, resulting in depressive symptoms (41).

The results also indicated that emotional abuse and neglect of childhood and parental rearing were significant predictors of depressive symptoms in only children and non-only children, which is following previous studies (42, 43). These findings highlight the protective role of the father’s emotional warmth and the negative impacts of the father’s rejection and the mother’s overprotection on only-child depressive symptoms. On the one hand, the interaction between parents and only children is more frequent than with non-only children. The only child is the center of the family and intra-family relationships would revolve around him/her (26). In this case, unbalanced family function directly affected the development of the only child (39). On the other hand, only children tend to rely on their parents, while adolescents in the traditional multi-child family prefer to spend time with their siblings and peers (26). Due to the increased interaction with and dependence on parents, only children may suffer from higher depressive symptoms when they experienced negative parental rearing styles such as rejection and overprotection. In most cultures, fathers tend to be strict and authoritarian with their children, which could result in more rejections. This parental rejection might affect their children’s depressive symptoms by lowering their self-esteem and making them more psychologically inflexible (44). Furthermore, father’s rather than mother’s emotional warmth was a significant protective factor for depressive symptoms in both only and non-only children, which was slightly different from some previous researches that indicated both father’s and mother’s emotional warmth would protect children from depression (45, 46). Nevertheless, other researches highlighted that more father’s emotional warmth was related to less depressive symptoms in adolescents, especially in boys (47, 48). However, in the real context of external stressors, adolescents tend to view the quality of their relationship with fathers in a more negative way than with mothers (49).

A key strength of the present study is the comprehensive and systematic measurements of childhood trauma and parental rearing style in adolescents. They were asked to evaluate the rearing styles of their father and mother separately. Furthermore, when exploring the predictive effect of parental factors on adolescent depressive symptoms, the only-child/non-only-child family infrastructure is more important than other family factors. The results also suggest that under the multi-child policy in China, counselors and educators may have a positive impact on the mental health of adolescents by promoting fathers to express warmth and acceptance toward their children.

There were several limitations in this study. Firstly, this was a cross-sectional study, which means it could only observe associations or correlations between variables at a specific point in time. It could not establish any causal relationships based on these observations. Secondly, the data were collected only in Shanghai, a Chinese metropolis. The results may not be extrapolated nationwide. Thirdly, this study did not take into account some demographic variables such as family socio-economic status. Previous studies found that lower social classes reported more overprotection and rejection (50). These factors might affect the study results and could be further explored in the future. Fourthly, stressful life events, the birth order of children, the age of other children at birth, and the gender of other children in non-only-child families were not taken into consideration, which may also have impacts on depressive symptoms in non-only children (51) and be worth exploring in the future.

## 5. Conclusion

In summary, the findings showed that girls and non-only children reported more depressive symptoms, while boys and non-only children perceived more childhood trauma and negative rearing styles. Emotional dimensions including emotional abuse, emotional neglect, and the father’s emotional warmth predicted depressive symptoms in both only children and non-only children. Moreover, the father’s rejection and the mother’s overprotection were related to only-child depressive symptoms. These findings call on parents to pay attention to their impacts on only children and attach great importance to non-only children’s mental health, as well as to devote more emotional care and support.

## Data availability statement

The raw data supporting the conclusions of this article will be made available by the authors, without undue reservation.

## Ethics statement

The studies involving human participants were reviewed and approved by Ethics Committee of Shanghai Mental Health Center. Written informed consent from the participants’ legal guardian/next of kin was not required to participate in this study in accordance with the national legislation and the institutional requirements.

## Author contributions

EC and YX designed the study. JC, YuL, YaL, and RC participated in the data collection. YZ and XH analyzed the data and wrote the manuscript. JC, EC, and YX assisted in manuscript revision. All authors have read and agreed to the published version of the manuscript.

## Funding

This study was supported by Shanghai Social Science Planning Project, Grant Number: 2019BSH012.

## Conflict of interest

The authors declare that the research was conducted in the absence of any commercial or financial relationships that could be construed as a potential conflict of interest.

## Publisher’s note

All claims expressed in this article are solely those of the authors and do not necessarily represent those of their affiliated organizations, or those of the publisher, the editors and the reviewers. Any product that may be evaluated in this article, or claim that may be made by its manufacturer, is not guaranteed or endorsed by the publisher.
