# Supplementary material for: The role of only-child status in the effect of childhood trauma and parental rearing style on depressive symptoms in Shanghai adolescents
Source: Front Psychiatry. 2023 Jun 21;14:1196569. doi: 10.3389/fpsyt.2023.1196569 (PMC10320292; doi:10.3389/fpsyt.2023.1196569)
Supplement: Supplementary file 1 [file Table_1.docx]

**Supplementary materials**

**Table S1.** **Gender and only-child/non-only-child differences in childhood trauma, parental rearing style, and depressive symptoms**

|  | **Total**  **(*n* = 4576)** | **Boys**  **(*n* = 2325)** | **Girls**  **(*n* = 2251)** | ***p*** | **Only-child**  **(*n* = 3236)** | **Non-only child**  **(*n* = 1340)** | ***p*** |
| --- | --- | --- | --- | --- | --- | --- | --- |
| EA | 6.70 ± 2.48 | 6.60 ± 2.38 | 6.81 ± 2.57 | 0.01^**^ | 6.65 ± 2.41 | 6.83 ± 2.64 | 0.03^*^ |
| PA | 5.54 ± 1.56 | 5.66 ± 1.72 | 5.42 ± 1.36 | 0.00^**^ | 5.51 ± 1.49 | 5.63 ± 1.72 | 0.02^*^ |
| SA | 5.11 ± 0.87 | 5.15 ± 1.05 | 5.08 ± 0.64 | 0.01^**^ | 5.11 ± 0.82 | 5.14 ± 1.00 | 0.29 |
| EN | 8.93 ± 3.66 | 8.97 ± 3.67 | 8.89 ± 3.65 | 0.48 | 8.71 ± 3.59 | 9.45 ± 3.77 | 0.00^**^ |
| PN | 6.86 ± 2.37 | 6.89 ± 2.37 | 6.82 ± 2.37 | 0.29 | 6.76 ± 2.30 | 7.09 ± 2.51 | 0.00^**^ |
| Rejection (F) | 7.99 ± 2.68 | 8.18 ± 2.77 | 7.81 ± 2.57 | 0.00^**^ | 7.94 ± 2.63 | 8.13 ± 2.80 | 0.03^*^ |
| Rejection (M) | 8.03 ± 2.65 | 8.06 ± 2.63 | 7.99 ± 2.68 | 0.39 | 7.95 ± 2.59 | 8.20 ± 1.98 | 0.01^**^ |
| Emotional warmth (F) | 22.15 ± 4.90 | 22.22 ± 4.85 | 22.08 ± 4.95 | 0.33 | 22.45 ± 4.76 | 21.43 ± 5.16 | 0.00^**^ |
| Emotional warmth (M) | 22.74 ± 4.54 | 22.85 ± 4.53 | 22.63 ± 4.54 | 0.10 | 23.04 ± 4.36 | 22.03 ± 4.87 | 0.00^**^ |
| Overprotection (F) | 15.72 ± 4.05 | 15.89 ± 3.95 | 15.54 ± 4.11 | 0.00^**^ | 15.71 ± 4.03 | 15.73 ± 4.09 | 0.91 |
| Overprotection (M) | 16.37 ± 4.22 | 16.45 ± 4.01 | 16.28 ± 4.43 | 0.18 | 16.38 ± 4.19 | 16.34 ± 4.29 | 0.81 |

Note: EA = Emotional abuse; PA = Physical abuse; SA = Sexual abuse; EN = Emotional neglect; PN = Physical neglect; F = Father; M = Mother.

^*^*p* < 0.05.

^**^*p* < 0.01.

**Table S2. Correlations between childhood trauma, parental rearing style, and depressive symptoms**

|  | **1** | **2** | **3** | **4** | **5** | **6** | **7** | **8** | **9** | **10** | **11** | **12** |
| --- | --- | --- | --- | --- | --- | --- | --- | --- | --- | --- | --- | --- |
| 1. EA | − |  |  |  |  |  |  |  |  |  |  |  |
| 2. PA | 0.53^**^ | − |  |  |  |  |  |  |  |  |  |  |
| 3. SA | 0.28^**^ | 0.48^**^ | − |  |  |  |  |  |  |  |  |  |
| 4. EN | 0.44^**^ | 0.28^**^ | 0.11^**^ | − |  |  |  |  |  |  |  |  |
| 5. PN | 0.34^**^ | 0.29^**^ | 0.19^**^ | 0.47^**^ | − |  |  |  |  |  |  |  |
| 6. Rejection (F) | 0.54^**^ | 0.44^**^ | 0.20^**^ | 0.39^**^ | 0.31^**^ | − |  |  |  |  |  |  |
| 7. Rejection (M) | 0.59^**^ | 0.44^**^ | 0.22^**^ | 0.43^**^ | 0.34^**^ | 0.76^**^ | − |  |  |  |  |  |
| 8. Emotional warmth (F) | − 0.44^**^ | − 0.25^**^ | − 0.07^**^ | − 0.58^**^ | − 0.35^**^ | − 0.48^**^ | − 0.42^**^ | − |  |  |  |  |
| 9. Emotional warmth (M) | − 0.44^**^ | − 0.25^**^ | − 0.08^**^ | − 0.59^**^ | − 0.36^**^ | − 0.40^**^ | − 0.49^**^ | 0.87^**^ | − |  |  |  |
| 10. Overprotection (F) | 0.33^**^ | 0.21^**^ | 0.10^**^ | 0.23^**^ | 0.16^**^ | 0.55^**^ | 0.46^**^ | − 0.28^**^ | − 0.27^**^ | − |  |  |
| 11. Overprotection (M) | 0.37^**^ | 0.22^**^ | 0.09^**^ | 0.26^**^ | 0.18^**^ | 0.47^**^ | 0.57^**^ | − 0.30^**^ | − 0.32^**^ | 0.85^**^ | − |  |
| 12. Depressive symptoms | 0.56^**^ | 0.33^**^ | 0.17^**^ | 0.50^**^ | 0.34^**^ | 0.47^**^ | 0.50^**^ | − 0.54^**^ | − 0.53^**^ | 0.35^**^ | 0.38^**^ | − |

EA = Emotional abuse; PA = Physical abuse; SA = Sexual abuse; EN = Emotional neglect; PN = Physical neglect; F = Father; M = Mother.

^*^*p* < 0.05.

^**^*p* < 0.01.
